# Supplementary material for: Regional hypothermia improves gastric microcirculatory oxygenation during hemorrhage in dogs
Source: PLoS One. 2019 Dec 10;14(12):e0226146. doi: 10.1371/journal.pone.0226146 (PMC6903746; doi:10.1371/journal.pone.0226146)
Supplement: S1 Table — Oral mucosal temperature in all experimental groups. Data are presented as absolute values, mean ± SEM, n = 6, * = p < 0.05 vs. baseline, # = p < 0.05 vs. respective normothermic control group during physiological conditions (HT-N vs. NT-N) and hemorrhagic shock (HT-H vs. NT-H), § = p < 0.05 vs. respective normovolemic control group during normothermic conditions (NT-H vs. NT-N) and hypothermia (HT-H vs. HT-N). 2-way ANOVA for repeated measurements followed by Bonferroni post hoc test. (DOCX) [file pone.0226146.s001.docx]

| parameter | group | 00:30 | | | | 01:00 | | | | 01:30 | | | | 02:00 | | | | 02:30 | | | | 03:00 | | | |
| --- | --- | --- | --- | --- | --- | --- | --- | --- | --- | --- | --- | --- | --- | --- | --- | --- | --- | --- | --- | --- | --- | --- | --- | --- | --- |
| [hh:mm] |  |  |  |  |  |  |  |  |  |  |  |  |  |  |  |  |  |  |  |  |  |  |  |  |  |
| oral Temp. [°C] | NT-N | 37.3 | ± | 0.3 |  | 37.3 | ± | 0.4 |  | 37.1 | ± | 0.4 |  | 37.2 | ± | 0.5 |  | 37.3 | ± | 0.4 |  | 37.5 | ± | 0.4 |  |
|  | HT-N | 37.7 | ± | 0.2 |  | 33.9 | ± | 0.2 | *# | 33.7 | ± | 0.2 | *# | 33.9 | ± | 0.1 | *# | 33.7 | ± | 0.2 | *# | 33.7 | ± | 0.2 | *# |
|  | NT-H | 37.8 | ± | 0.2 |  | 37.6 | ± | 0.2 |  | 37.6 | ± | 0.2 |  | 37.8 | ± | 0.2 | § | 37.9 | ± | 0.2 | § | 37.7 | ± | 0.2 |  |
|  | HT-H | 37.9 | ± | 0.3 |  | 33.8 | ± | 0.1 | *# | 34.0 | ± | 0.1 | *# | 33.6 | ± | 0.2 | *# | 34.0 | ± | 0.1 | *# | 33.9 | ± | 0.1 | *# |
