# Supplementary material for: Regional hypothermia improves gastric microcirculatory oxygenation during hemorrhage in dogs
Source: PLoS One. 2019 Dec 10;14(12):e0226146. doi: 10.1371/journal.pone.0226146 (PMC6903746; doi:10.1371/journal.pone.0226146)
Supplement: S2 Table — Arterial oxygen partial pressure (PaO2), carbon dioxide partial pressure (PaCO2), haematocrit (Hct), pH, bicarbonate (HCO3-), and lactate plasma levels. Data are presented as absolute values, mean ± SEM, n = 6, * = p < 0.05 vs. baseline, # = p < 0.05 vs. respective normothermic control group during physiological conditions (HT-N vs. NT-N) and hemorrhagic shock (HT-H vs. NT-H), § = p < 0.05 vs. respective normovolemic control group during normothermic conditions (NT-H vs. NT-N) and hypothermia (HT-H vs. HT-N). 2-way ANOVA for repeated measurements followed by Bonferroni post hoc test. (DOCX) [file pone.0226146.s002.docx]

| parameter | group | 00:30 | | | | 01:00 | | | | 01:30 | | | | 02:00 | | | | 02:30 | | | | 03:00 | | | |
| --- | --- | --- | --- | --- | --- | --- | --- | --- | --- | --- | --- | --- | --- | --- | --- | --- | --- | --- | --- | --- | --- | --- | --- | --- | --- |
| [hh:mm] |  |  |  |  |  |  |  |  |  |  |  |  |  |  |  |  |  |  |  |  |  |  |  |  |  |
| pCO_2_ [mmHg] | NT-N | 37.3 | ± | 1.2 |  | 38.0 | ± | 0.8 |  | 38.3 | ± | 0.8 |  | 37.7 | ± | 0.8 |  | 37.9 | ± | 0.8 |  | 37.8 | ± | 0.6 |  |
|  | HT-N | 37.4 | ± | 1.0 |  | 37.1 | ± | 0.8 |  | 38.3 | ± | 0.9 |  | 38.9 | ± | 1.0 | * | 39.0 | ± | 0.8 | * | 38.3 | ± | 0.7 |  |
|  | NT-H | 37.9 | ± | 1.1 |  | 37.5 | ± | 1.0 |  | 43.2 | ± | 1.5 | *§ | 41.8 | ± | 1.4 | *§ | 37.8 | ± | 0.5 |  | 37.1 | ± | 1.3 |  |
|  | HT-H | 37.2 | ± | 1.1 |  | 37.7 | ± | 1.1 |  | 43.5 | ± | 1.5 | *§ | 42.5 | ± | 1.4 | *§ | 37.6 | ± | 0.9 |  | 37.8 | ± | 0.8 |  |
| pO_2_ [mmHg] | NT-N | 136.7 | ± | 5.2 |  | 136.8 | ± | 5.1 |  | 138.3 | ± | 4.3 |  | 139.2 | ± | 5.1 |  | 140.8 | ± | 4.5 |  | 141.2 | ± | 4.7 |  |
|  | HT-N | 138.7 | ± | 5.7 |  | 140.2 | ± | 5.2 |  | 140.8 | ± | 5.2 |  | 143.0 | ± | 5.4 |  | 144.7 | ± | 5.6 | * | 148.8 | ± | 5.3 | * |
|  | NT-H | 137.5 | ± | 7.6 |  | 140.3 | ± | 6.6 |  | 128.3 | ± | 7.1 | * | 138.8 | ± | 5.4 |  | 150.5 | ± | 5.8 | * | 147.8 | ± | 4.8 | * |
|  | HT-H | 137.8 | ± | 5.9 |  | 139.2 | ± | 5.0 |  | 130.2 | ± | 5.0 | * | 135.2 | ± | 3.9 |  | 144.5 | ± | 4.9 | * | 146.0 | ± | 4.4 | * |
| pH | NT-N | 7.38 | ± | 0.01 |  | 7.37 | ± | 0.01 |  | 7.36 | ± | 0.01 | * | 7.36 | ± | 0.01 | * | 7.36 | ± | 0.01 | * | 7.36 | ± | 0.01 | * |
|  | HT-N | 7.38 | ± | 0.01 |  | 7.38 | ± | 0.01 |  | 7.38 | ± | 0.01 |  | 7.37 | ± | 0.01 |  | 7.36 | ± | 0.01 | * | 7.36 | ± | 0.01 |  |
|  | NT-H | 7.38 | ± | 0.01 |  | 7.38 | ± | 0.01 |  | 7.31 | ± | 0.01 | * | 7.32 | ± | 0.01 | * | 7.36 | ± | 0.01 | * | 7.38 | ± | 0.02 |  |
|  | HT-H | 7.38 | ± | 0.01 |  | 7.38 | ± | 0.01 |  | 7.31 | ± | 0.02 | * | 7.31 | ± | 0.02 | * | 7.36 | ± | 0.01 | * | 7.37 | ± | 0.01 | * |
| HCO_3_^-^ [mmol·l^-1^] | NT-N | 21.3 | ± | 0.3 |  | 21.2 | ± | 0.2 |  | 20.8 | ± | 0.2 |  | 20.6 | ± | 0.4 | * | 20.6 | ± | 0.4 | * | 20.6 | ± | 0.3 | * |
|  | HT-N | 21.1 | ± | 0.5 |  | 21.1 | ± | 0.4 |  | 21.6 | ± | 0.4 |  | 21.5 | ± | 0.4 |  | 21.3 | ± | 0.4 |  | 21.1 | ± | 0.3 |  |
|  | NT-H | 21.8 | ± | 0.3 |  | 21.3 | ± | 0.2 |  | 21.0 | ± | 0.3 | * | 20.7 | ± | 0.2 | * | 20.8 | ± | 0.2 | * | 21.0 | ± | 0.3 | * |
|  | HT-H | 21.3 | ± | 0.3 |  | 21.3 | ± | 0.3 |  | 20.9 | ± | 0.4 |  | 20.8 | ± | 0.4 |  | 20.7 | ± | 0.3 |  | 20.8 | ± | 0.3 |  |
| BE [mmol·l^-1^] | NT-N | -2.8 | ± | 0.2 |  | -3.0 | ± | 0.2 |  | -3.6 | ± | 0.3 | * | -3.8 | ± | 0.5 | * | -3.8 | ± | 0.5 | * | -3.8 | ± | 0.4 | * |
|  | HT-N | -3.0 | ± | 0.6 |  | -3.1 | ± | 0.6 |  | -2.5 | ± | 0.6 |  | -2.9 | ± | 0.5 |  | -3.1 | ± | 0.5 |  | -3.2 | ± | 0.4 |  |
|  | NT-H | -2.3 | ± | 0.3 |  | -2.8 | ± | 0.3 |  | -4.2 | ± | 0.3 | * | -4.3 | ± | 0.3 | * | -3.6 | ± | 0.3 | * | -3.1 | ± | 0.5 | * |
|  | HT-H | -2.8 | ± | 0.4 |  | -2.9 | ± | 0.4 |  | -4.3 | ± | 0.5 | * | -4.3 | ± | 0.5 | * | -3.6 | ± | 0.5 | * | -3.4 | ± | 0.4 |  |
| lactate [mmol·l^-1^] | NT-N | 0.9 | ± | 0.1 |  | 1.1 | ± | 0.1 |  | 1.5 | ± | 0.2 | * | 1.7 | ± | 0.3 | * | 1.8 | ± | 0.3 | * | 1.8 | ± | 0.3 | * |
|  | HT-N | 0.9 | ± | 0.1 |  | 0.9 | ± | 0.0 |  | 1.0 | ± | 0.1 |  | 1.1 | ± | 0.1 |  | 1.1 | ± | 0.1 |  | 0.9 | ± | 0.1 |  |
|  | NT-H | 1.0 | ± | 0.1 |  | 1.2 | ± | 0.1 |  | 1.6 | ± | 0.1 | * | 1.6 | ± | 0.2 | * | 1.4 | ± | 0.2 | * | 1.4 | ± | 0.2 | * |
|  | HT-H | 1.2 | ± | 0.3 |  | 1.3 | ± | 0.3 |  | 1.5 | ± | 0.3 |  | 1.4 | ± | 0.2 |  | 1.3 | ± | 0.2 |  | 1.2 | ± | 0.2 |  |
| Hb [g·100ml^-1^] | NT-N | 11.3 | ± | 0.5 |  | 11.3 | ± | 0.6 |  | 11.2 | ± | 0.6 |  | 11.2 | ± | 0.6 |  | 11.2 | ± | 0.6 |  | 11.2 | ± | 0.6 |  |
|  | HT-N | 11.1 | ± | 0.5 |  | 11.2 | ± | 0.5 |  | 11.1 | ± | 0.5 |  | 11.1 | ± | 0.6 |  | 11.1 | ± | 0.6 |  | 11.2 | ± | 0.5 |  |
|  | NT-H | 11.2 | ± | 0.5 |  | 11.0 | ± | 0.6 |  | 11.3 | ± | 0.5 |  | 11.2 | ± | 0.4 |  | 10.9 | ± | 0.5 |  | 11.2 | ± | 0.8 |  |
|  | HT-H | 11.3 | ± | 0.5 |  | 11.3 | ± | 0.6 |  | 11.5 | ± | 0.6 |  | 11.3 | ± | 0.5 |  | 11.1 | ± | 0.5 |  | 11.0 | ± | 0.5 |  |
